# Queen Charlotte's Hospital and Its Nursing Staff

**Published:** 1919-09-20

**Authors:** 


*To the Editor of* THE HOSPITAL.

### QUEEN CHARLOTTE'S HOSPITAL AND ITS NURSING STAFF.

*Nursing Hours—Question of Cleanliness—Cleaning Materials not Provided by Hospital—Defective Food Supply—Working Hours, 6.30 a.m. to 9.30 p.m.—Can Recreation have a Place in the Curriculum of the Pupil-midwife?*

*To the Editor of* THE HOSPITAL.

SIR,—The appeal of Queen Charlotte's Hospital for additional funds to meet increased expenditure includes also a sum estimated at £40,000 for schemes of extension and improvement. It is hoped and expected by those who for many years have materially assisted and watched the affairs of this hospital that among the improvements to be evolved some may be directed toward the general working conditions and the working hours of the nursing staff and the nurse-pupils. The physical and mental exhaustion which follows a normal day of work in the wards of this hospital—a day beginning at 6.30 A.M. and ending at 9.30 P.M., with a bare two hours off duty during that time for the pupils—is contrary to the common laws of humanity and in direct contrast to modern ideas governing women's labour in whatever direction it may lie. The existing thirteen hours of almost continuous duty might with advantage be reduced to a more moderate nine or even ten hours a day, and the working conditions so materially improved as to afford the pupils a better opportunity of theoretical study, so as to attend their class lectures with a lessened sense of physical oppression, and to have, what is of equal importance, greater opportunity of breathing the air of heaven. Recreation is neither asked nor expected for them: as all sensible students know, there can be no place for such in the curriculum of midwifery.

It is reported that at Queen Charlotte's Hospital the duty of surgically cleaning the lying-in wards to which they are attached is imposed upon the pupil-midwives. This consists of cleaning down the walls and furniture with antiseptic water and polishing the brass taps and various fittings, ward maids being responsible for the floor only. Cleaning materials are not well supplied by this hospital, and it is the general custom for the pupils to supply such cleaning materials as "Monkey Brand," "Vim," and "Brasso" from money out of their own pockets for the purpose of bringing their work up to the standard required by inspection.

The food supplied to the nursing staff leaves much to be desired, considering the arduous and tiring nature of their duties, and has to be supplemented by the pupils at near-by restaurants in off-duty times. Pupils sometimes spend from 10s. to 15s. per week upon supplementary subsistence, bread and marmalade on certain mornings being all that is supplied for breakfast. Add to this the high rate of laundry expenses by reason of always requiring two, and frequently three, dresses a week—the burden of this also borne by the pupils—and the fact that she pays down forty guineas on entering the hospital, a career at Queen Charlotte's Hospital becomes an expensive luxury.—Yours faithfully,

"INTERESTED."

